# Aneurysmal subarachnoid hemorrhage: factors associated with worse functional outcome

**DOI:** 10.1186/2197-425X-3-S1-A782

**Published:** 2015-10-01

**Authors:** L Sánchez Montori, J González Robledo, ME Pérez Losada, M Pérez Cheng, A Rodríguez Encinas

**Affiliations:** Intensive Care Department, University Hospital of Salamanca, Salamanca, Spain

## Introduction

Subarachnoid hemorrhage (SAH) is a devastating neurological emergency. Although it is a relatively common and potentially curable disease, it presents a high morbidity and mortality.

## Objectives

Describe the factors associated with worse functional outcome, determined by an Extended Glasgow Outcame Scale (GOSE) of 2-3, in patients with a spontaneous subarachnoid hemorrhage of aneurysmal origin in our center.

## Methods

Data from patients admitted to the ICU with SAH between January 2006 and December 2014 were retrospectively collected. We analyzed epidemiological variables, past history, severity scale scores (Glasgow Coma Scale at admission, Hunt-Hess, World Federation of Neurosurgical Societies Scale, Fisher and APACHE II), diagnostic methods, therapeutic management, complications, functional status at discharge and mortality. T test was used to compare means and logistic regression for assessing predictors. Data was analyzed with SPSS v19.

## Results

We included 151 patients, with a mortality rate of 17.2%. GOSE 2-3 was found in 28.8% and 4-5 in 71.2%. Patients with worse outcome were older [64.47 (SD 12.64) vs 56.92 (SD 12.93)], with higher APACHE II [20.42 (SD 6.85) vs 13.15 (SD 6.60)], worst GCS at admission [8 (SD 4.03) vs 13.2 (SD 3.01)], higher scores on the prognostic scales: Hunt-Hess IV-V (75% vS 16.8 %), Fischer IV (91.7% vs 57.3%), WFNS IV-V 80.5% vs 25.9%). These differences were not statistically significant, except for the GCS at admission. Complications were more frequent in the GOSE 2-3 group: hydrocephalus (72.2% vs 21.3%), intracranial hypertension (38.9% vs 7.9%) and rebleeding (19.4 % vs 4.5%) were statistically significant. Logistic regression analysis results are summarized at table 1:

**Table 1:** Logistic regression analysis.

VARIABLE	OR	CI 95% OR	p
Rebleeding	5.13	1.40 - 18.80	0,012
Hidrocephalus	9.58	3.94 - 23.29	< 0.001
Ventriculitis	2.56	0.35 - 18.90	0.365
Intracraneal hypertension	7.46	2.68 - 20.72	< 0.001
Delayed ischemic deficit	1.27	0.40 - 4.03	0.68
GCS 3-8	20.47	7.04 - 59.52	< 0.001
GCS 9-13	3.87	1.15 - 13.01	< 0.001

## Conclusions

SAH has a high morbidity and mortality. In our series, factors associated with worse functional outcome at discharge were GCS ≤13 at admission, presence of hydrocephalus, intracranial hypertension and rebleeding.

## References

[1] Steiner T, Juvela S, Unterberg A, Jung C, Forsting M, Rinkel G (2013). European Stroke Organization Guidelines for the Management of Intracranial Aneurysms and Subarachnoid Haemorrhage. Cerebrovascular Diseases.

[2] Michael N, Diringer , Thomas P, Bleck J, Claude Hemphill III, David Menon , Lori Shutter , Paul Vespa , Nicolas Bruder E, Sander Connolly , Giuseppe Citerio , Daryl Gress , Daniel Hänggi , Brian L. Hoh , Giuseppe Lanzino , Peter Le Roux , Alejandro Rabinstein , Erich Schmutzhard , Nino Stocchetti , Jose I. Suarez , Miriam Treggiari , Ming-Yuan Tseng , Mervyn DI, Vergouwen , Stefan Wolf , Gregory Zipfel : Critical Care Management of Patients Following Aneurysmal Subarachnoid Hemorrhage: Recommendations from the Neurocritical Care Society´s Multidisciplinary Consensus Conference. 10.1007/s12028-011-9605-9 21773873

